# Optic disc drusen mimicking Idiopathic Intracranial Hypertension (IIH): rely on ultrasound

**DOI:** 10.1186/s42466-021-00133-0

**Published:** 2021-06-14

**Authors:** Eleni Bakola, Dimitrios Alonistiotis, Chryssa Arvaniti, Stavroula Salakou, Niki Nana, Aikaterini Foska, Vasiliki Kotsali-Peteinelli, Konstantinos Voumvourakis, Georgios Tsivgoulis

**Affiliations:** 1 Second Department of Neurology, National and Kapodistrian University of Athens, School of Medicine, “Attikon” University Hospital, Rimini 1, Chaidari, 12462 Athens, Greece; 2 Second Department of Ophthalmology, National and Kapodistrian University of Athens, School of Medicine, “Attikon” University Hospital, Athens, Greece; 3 Department of Neurology, The University of Tennessee Health Science Center, Memphis, TN USA

**Keywords:** Idiopathic intracranial hypertension, Papilledema, Optic disc drusen, Transorbital ultrasound

## Abstract

Optic nerve ultrasound is an established routine supplementary diagnostic tool for idiopathic intracranial pressure but it can also be helpful in avoiding misdiagnoses. We describe a case of an obese 15- year-old girl with persistent headaches, fundoscopic findings suggesting papilledema, normal brain imaging who underwent two lumbar punctures with unremarkable cerebrospinal fluid findings before ultrasound revealed optic disc drusen as the cause of the optic disc elevation.

## Introduction

Identification of optic nerve head edema in a young woman with chronic headaches and normal brain imaging is highly suggestive of idiopathic intracranial hypertension (IIH) [1]. However, extensive work-up sometimes reveals alternative diagnoses.

## Methods

In this case report, we describe the diagnostic work-up of an obese 15 year old girl with persistent headaches and fundus findings suggesting papilledema.

## Case description

A 15-year-old girl with a BMI of 32 kg/m2 was referred to an outside Institution by an outpatient ophthalmologist due to abnormal fundus findings suggesting “papilledema” in order to rule out IIH. The girl complained about recurrent headaches over the past year without any other symptoms such as transient visual obscurations, pulsatile tinnitus, blurred vision or diplopia. Neurological examination and visual acuity were normal. Past medical history was unremarkable. Brain magnetic resonance imaging (MRI) and MR venography (MRV) were normal. She underwent lumbar puncture with normal cerebral spinal fluid (CSF) findings and opening pressure measured at 23 cm Η_2_Ο. All laboratory examinations were unremarkable. No diagnosis of IIH was made at that time.

**Fig. 1:**
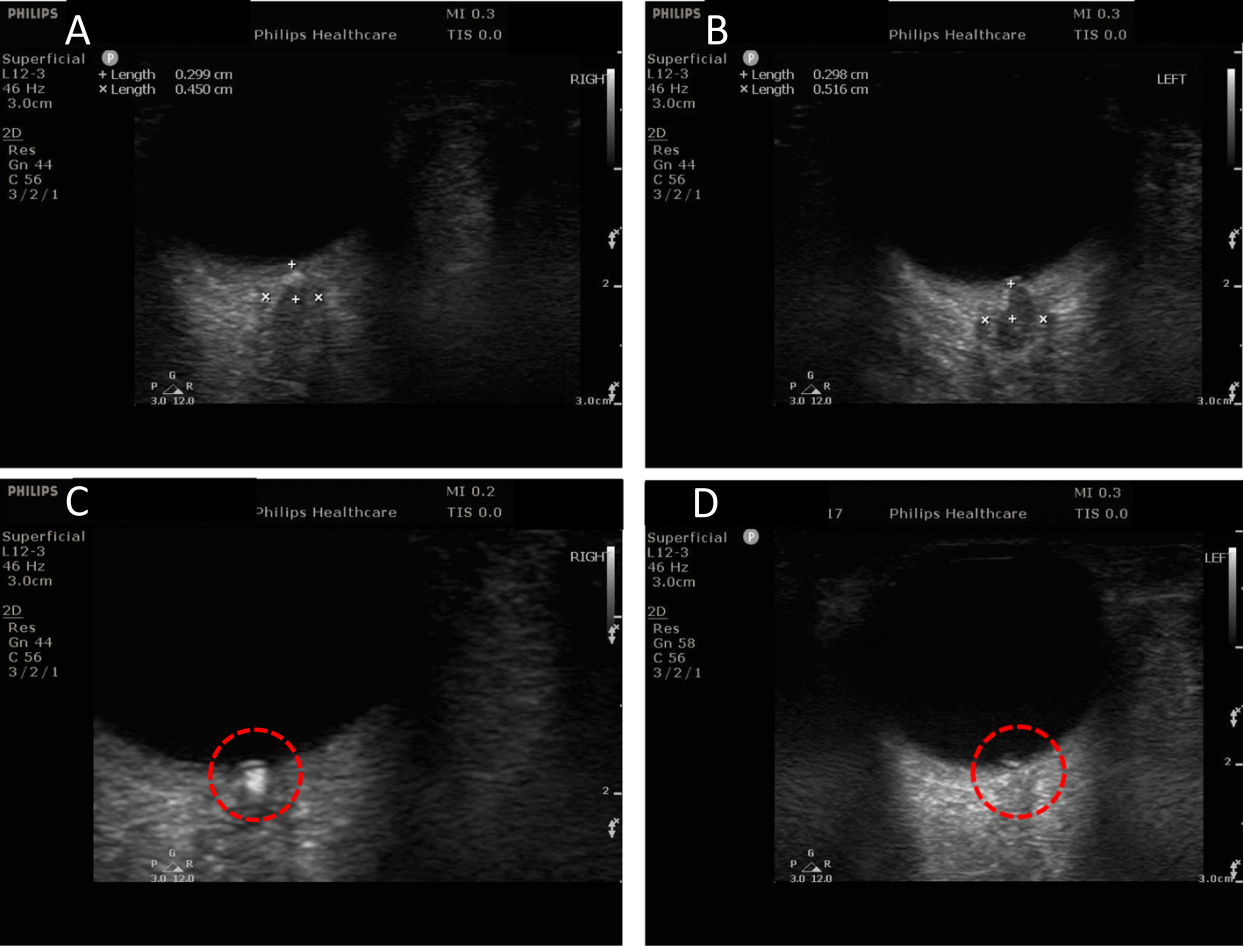
Transorbital Ultrasound Findings in suspected IIH revealing Optic Disc Drusen. B-Mode transorbital sonography ruled out enlargement of the optic nerve sheath diameter as measured 3 mm posterior to the globe (right 0.45 cm, left 0.52 cm, normal limits < 0.51 cm for European population [2]) due to underlying IIH (Panels **a** & **b**). Calcified optic disc drusen appeared as hyperechoic, highly reflective structures with posterior acoustic shadow (red-circle; Panels **c** & **d**)

Six months later, she was referred to our department because of persisting abnormal appearance of the optic nerves in the fundus examination interpreted again as papilledema and because of recurring headaches. Once again, she underwent lumbar puncture with normal CSF composition and normal CSF opening pressure (26 cm Η_2_0). An ultrasonography of the optic nerve sheath diameter (ONSD) was performed and -correlating with the measured CSF opening pressure- it was found within normal limits (right 4.5 mm, left 5.2 mm) (Fig. 1; Panels a & b) [2]. Furthermore, ultrasound was able to detect hyperechoic, highly reflective structures with posterior acoustic shadow at both optic nerve heads suggesting the presence of calcified optic disc drusen (ODD) (Fig. 1; Panels c & d).

**Fig. 2:**
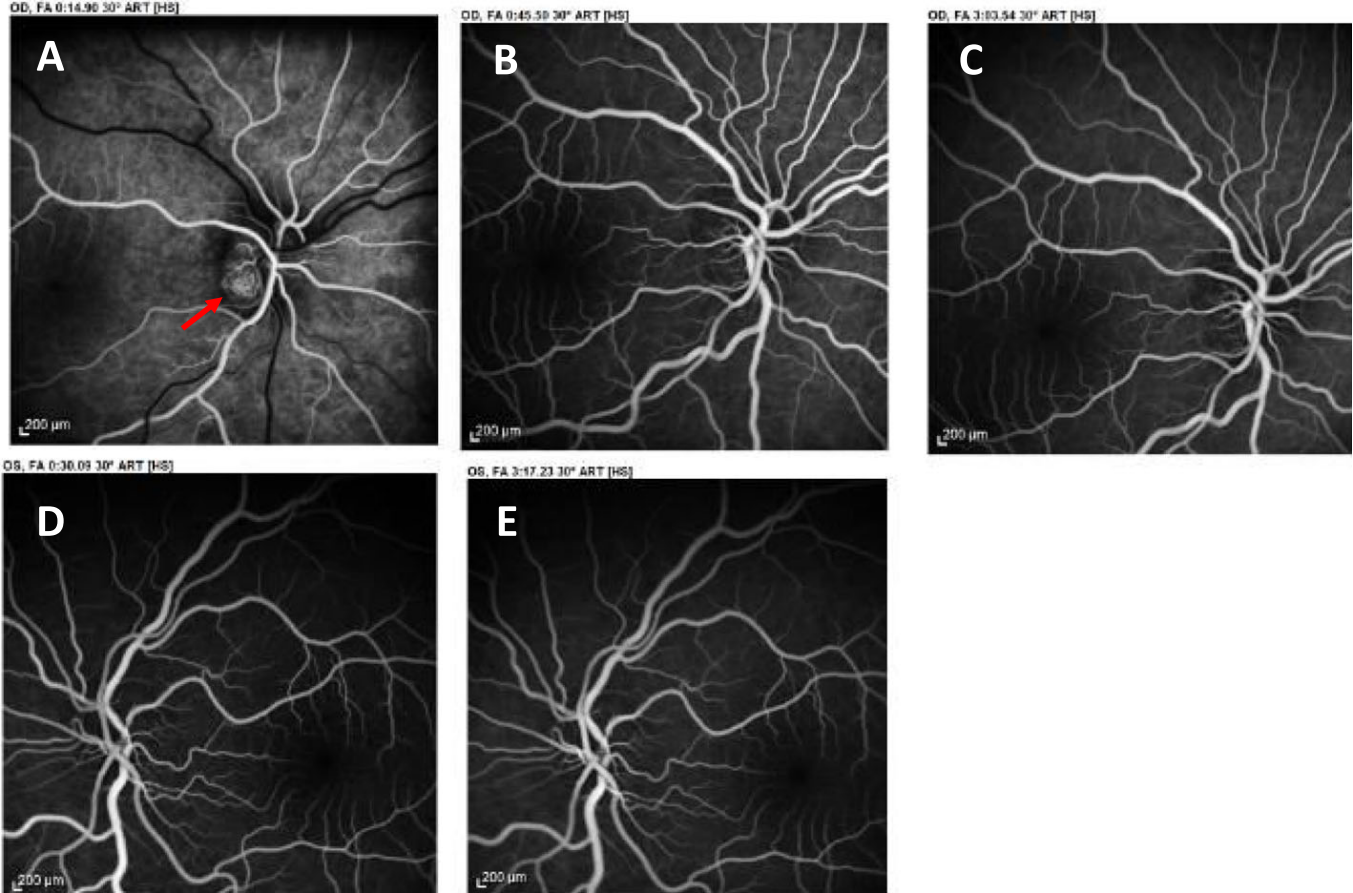
Fluorescein angiography reveals Optic Disc Drusen ruling out IIH**.** Arterial phase of FA revealing early nodular staining of the disc due to presence of buried ODD (red arrow) - right eye (Panel **a**). No early or late fluorescein leakage suggesting papilledema due to IIH as noted (right - Panel **b** & **c**: mid-and late-phase angiography; left- Panel **d** & **e**: mid-and late-phase angiography)

With regard to that finding, fluoroscein angiography was performed and revealed ODD as the cause of the optic disc elevation which was falsely interpreted as papilledema and definitely ruled out IIH (Fig. 2). The patient was diagnosed with tension type headache and amitriptyline (10 mg qd) was prescribed.

## Discussion

IIH is a disorder of unknown etiology characterized by an increase of intracranial pressure (ICP) with typical corresponding neuroimaging findings on brain MRI & MRV [3]. Over the last years, sonographic assessment of papilledema and enlarged ONSD has become a valuable non-invasive adjunct method to detect and monitor elevated ICP in patients with IIH [4].

However, when predominately relying on interpretation of fundus findings the diagnosis of IIH can be challenging since not all optic disc swelling is due to papilledema. Examination of the ocular fundus is often misinterpreted especially in female obese headache patients resulting in invasive and costly tests, medication use and in some cases in unnecessary aggressive treatments. According to a large retrospective study overdiagnosis of IIH was documented in 34/86 patients (39.5%) with presumed IIH, especially due to errors committed in fundus evaluation mainly in obese women in childbearing age. ODD was responsible for 2/34 misdiagnoses (6%) [5].

ODD are acellular deposits of calcium, amino and nucleic acids, and mucopolysaccharides in the optic nerve head with a reported prevalence in adults of 1.8 and 1% in children [6, 7]. ODD are bilateral in most cases. Patients with ODD are usually asymptomatic, and are often coincidentally diagnosed. ODD may be buried or superficial. Buried ODD are more prevalent in children and produce the funduscopic appearance of an elevated optic nerve head which is often mistaken for true papilledema [8].

Ocular ultrasound is very useful in the detection of ODD including buried drusen. It is a reliable, inexpensive and fast diagnostic tool. B-scan ultrasound is considered diagnostic if there is an area of hyperreflectivity present at the nerve head. ODD also cause acoustic shadowing of posterior structures [9].

In conclusion, the routine use of sonographic measurements of ONSD values is useful in detecting raised ICP in patients with presumed IIH as it easily detects papilledema and enlarged ONSD in IIH. Furthermore, the diagnostic utility of optic nerve sonography may further increase since it may reveal ODD as the underlying cause of optic disc elevation in fundoscopy, avoid IIH misdiagnosis and prevent an extensive, invasive and unnecessary diagnostic work-up.

## Data Availability

All data are presented in the manuscript.

## Abbreviations

IIH: Idiopathic intracranial pressure
ODD: Optic disc drusen
ICP: Intracranial pressure
ONSD: Optic nerve sheath diameter
MRI: Magnetic resonance imaging
MRV: Magnetic resonance venography
CSF: Cerebral spinal fluid
